# Interaction between *Vibrio mimicus* and *Acanthamoeba castellanii*

**DOI:** 10.1111/j.1758-2229.2009.00129.x

**Published:** 2010-02

**Authors:** Hadi Abd, Soni Priya Valeru, Susan Marouf Sami, Amir Saeed, Saumya Raychaudhuri, Gunnar Sandström

**Affiliations:** 1 Swedish Institute for Infectious Disease Control, Centre for Microbiological PreparednessSE-17182 Solna, Sweden; 2 Karolinska Institute, Department of Laboratory Medicine, Division of Clinical Microbiology, Karolinska University HospitalHuddinge, SE-141 86 Stockholm, Sweden; 3 Institute of Microbial Technology, CSIRSector 39-A, Chandigarh, 160036, India

## Abstract

*Vibrio mimicus* is a Gram-negative bacterium, which causes gastroenteritis and is closely related to *Vibrio cholerae*. The environmental reservoir of this bacterium is far from defined. *Acanthamoeba* as well as *Vibrio* species are found in diverse aquatic environments. The present study was aimed to investigate the ability of *A. castellanii* to host *V. mimicus*, the role of bacterial protease on interaction with *A. castellanii* and to disclose the ability of cysts to protect intracellular *V. mimicus*. Co-cultivation, viable counts, gentamicin assay, electron microscopy and statistical analysis showed that co-cultivation of wild type and *luxO* mutant of *V. mimicus* strains with *A. castellanii* did not inhibit growth of the amoeba. On the other hand co-cultivation enhanced growth and survival of *V. mimicus* strains. *Vibrio mimicus* showed intracellular behaviour because bacteria were found to be localized in the cytoplasm of amoeba trophozoites and remain viable for 14 days. The cysts protected intracellular *V. mimicus* from high level of gentamicin. The intracellular growth of *V. mimicus* in *A. castellanii* suggests a role of *A. castellanii* as a host for *V. mimicus*.

## Introduction

*Vibrio mimicus* is a Gram-negative bacterium that is an autochthonous member of diverse aquatic environments and associated with freshwater prawns, raw fishes in Bangladesh and Japan (Chowdhury *et al.*, 1989). As a consequence ingestion of raw or undercooked seafood turns out to be a primary cause of *V. mimicus* gastroenteritis (Chitov *et al.*, 2009).

Bacterial growth and survival are subject to constraint by bacterivores such as free-living amoebae (Matz *et al.*, 2005). The free-living amoebae including *Acanthamoeba* species are commonly found in various natural sources such as soil, freshwater and salt water (Martinez and Visvesvara, 1997). The life cycle of *Acanthamoeba* consists of an actively feeding trophozoite and a dormant cyst that occurs under adverse environmental conditions. In their natural environment, amoebae coexist with bacteria, thus exert a strong influence on the survival and behaviour of microbial community. It is well known that *Acanthamoeba* species are environmental hosts of many bacteria (Winiecka-Krusnell and Linder, 2001). Recent studies have evidenced that *Vibrio cholerae* O1 and O139 are capable of surviving and replicating inside *Acanthamoeba castellanii* findings that render *V. cholerae* as a facultative intracellular bacterium (Abd *et al.*, 2005; Abd *et al.*, 2007; Abd *et al.*, 2009).

*Vibrio mimicus* shares similar properties with *V. cholerae* such as existence of virulence associated genes, namely cholera toxin as well as toxin co-regulated pilus genes (Shinoda *et al.*, 2004) and both species possess LuxO protein that regulates protease activity (Sultan *et al.*, 2006). Protease is a potential virulence factor of *V. mimicus*, a bacterium of medical importance among *Vibrio* species because it causes gastroenteritis and is associated with wound and middle ear infections (Davis *et al.*, 1981; Shandera *et al.*, 1983).

*Vibrio mimicus* shares similar properties with *V. cholerae* O1 and O139 being able to survive in *A. castellanii* (Abd *et al.*, 2007; Abd *et al.*, 2009); thus we aimed to study interaction of *A. castellanii* with *V. mimicus* to disclose ability of *V. mimicus* to survive amoebic phagocytosis and to grow inside *A. castellanii*; role of *A. castellanii* as host for *V. mimicus*; encystations of the amoebae carrying intracellular *V. mimicus* as an amoebic vital process and as a process to protect the intracellular *V. mimicus* from antibiotic killing and to find out whether protease affects growth of *A. castellanii* or not.

## Results and discussion

### Effect of *V. mimicus* on the growth of *A. castellanii*

Cell counts of viable *A. castellanii* in the absence and the presence of a wild strain of *V. mimicus* as well as its *luxO* mutant strain were performed to study the ability of amoebae to grow alone and during co-cultivation. Moreover, experiments were designed to find out whether protease affects the growth of *A. castellanii* or not because the *luxO* mutant strain has increased protease activity compared with the wild *V. mimicus*.

**Fig. 1:**
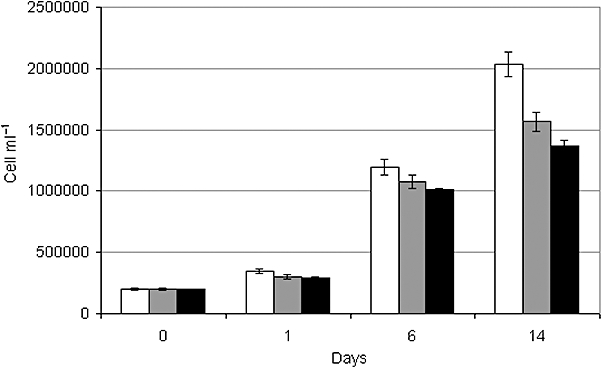
Growth of *A. castellanii*. White staples indicate growth of *A. castellanii* in the absence of bacteria, grey in presence of wild type *V. mimicus* CS-5, and black in the presence of *luxO* disruptant mutant LODC 5. Data indicate mean ± SD values of three repeated experiments.

Growth of *A. castellanii* in the presence or absence of *V. mimicus* strain CS-5 and its isogenic mutant LODC-5 were studied by viable amoeba cell counts. The initial concentration of the amoebae (trophozoites and cysts) was 2 × 10^5^ cells ml^−1^, which increased 10-fold in the absence of bacteria. Eightfold respective sevenfold increases of amoebae were estimated in the presence of wild or mutant strain after 14 days (Fig. 1). χ^2^ test did not show statistically significant difference in the growth of *A. castellanii* in the presence or absence of *V. mimicus* strain CS-5 or its mutant LODC-5 (*P* = 0.93).

The result showed that *A. castellanii* grew onefold less in the presence of *luxO* disruptant strain than the wild *V. mimicus* but the difference in growth of amoebae in presence of wild bacteria or its mutant was not statistically significant (*P* = 0.99). Sultan and colleagues found that LuxO mutant possessed significantly higher protease activity than the wild strain during the log phase of growth. But no significant differences could be seen in protease activity of the strains in stationary phase or after 24 h of growth (Sultan *et al.*, 2006).

Protease activity of wild and *luxO* mutant was investigated. The analysis showed that wild-type and mutant *V. mimicus* strains in the presence of amoeba did not have any significant differences in protease activity (*P* of *t*-test was 0.34) under the stationary phase of bacterial growth (data not shown). This may explain why the growth of amoebae in the presence of wild or mutant *V. mimicus* strains was not markedly affected.

### Effect of *A. castellanii* on the growth of *V. mimicus* strains

Viable counts of wild *V. mimicus* or its *luxO* mutant strain in the absence or the presence of *A. castellanii* was adjusted to study growth of the bacteria alone and in co-culture in order to investigate if the amoebae enhanced or inhibited growth of *V. mimicus*.

**Fig. 2:**
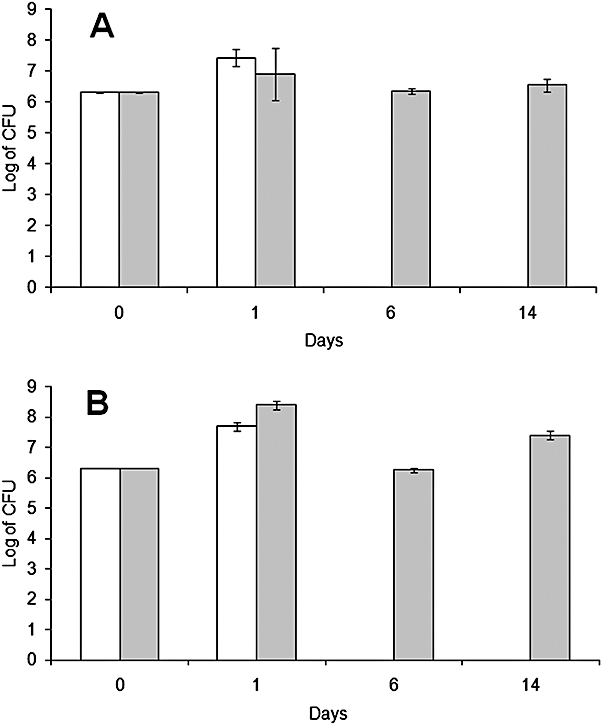
Growth of *V. mimicus*. (A) Wild type *V. mimicus* CS-5 and (B) *luxO* disruptant mutant LODC 5. White staples indicate alone cultured bacteria and the grey co-cultured with *A. castellanii*. Data indicate mean values ± SD of three repeated experiments.

The results showed that wild *V. mimicus* and its mutant strain in the presence of *A. castellanii* increased 10-fold and 100- fold, respectively, after 1 day of incubation and bacteria survived for more than 2 weeks (Fig. 2). Viable counts of all *V. mimicus* strains in the absence of amoebae increased 10-fold during the first day followed by a decrease to 0.0 cfu ml^−1^ on day 6 (Fig. 2). Student's *t*-test showed a statistically significant difference in the growth of wild and mutant *V. mimicus* in the presence or the absence of *A. castellanii* (*P*-values were 0.0001 and 0.00003).

It is interesting to note that the growth of *V. mimicus* strains was enhanced in the presence of *A. castellanii* whereas the growth of bacteria in the absence of amoebae has decreased to non-detectable levels. However, growth rate of the mutant strain has increased 10-fold more than the wild strain from day 1 to day 14 (Fig. 2). This may explain why *A. castellanii* grew onefold less in the presence of *luxO* disruptant strain than the wild *V. mimicus* (Fig. 1).

We observed that the growth of amoebae was not inhibited in the presence of wild or *luxO* mutant of *V. mimicus* indicates that protease did not exert a deleterious effect on the growth of amoeba. It is noteworthy that the protease of *Pseudomonas aeruginosa* had no effect on the growth of *A. castellanii* cells, which were killed by type III-secreted proteins (Abd *et al.*, 2008). On the contrary, PrtV, an extracellular protease of *V. cholerae*, is necessary for killing of the worm *Caenorhabditis elegans* (Vaitkevicius *et al.*, 2006).

### Intracellular growth, survival and localization of *V. mimicus*

Samples were taken from co-culture flasks and viable counts of intracellular *V. mimicus* were investigated. Gentamicin and sodium deoxycholate treatment were utilized to kill extracellular bacteria and to permeabilize amoeba cells in order to release the intracellular bacteria in agar plates. The result showed that no viable bacteria were detected in the supernatants after gentamicin treatment compared with the intracellular bacteria, which grew to 3.0 × 10^2^ ± 1.0 × 10^2^ cfu ml^−1^ after 2 h of co-cultivation, 6.0 × 10^2^ ± 2.0 × 10^2^ cfu ml^−1^ after 4 h and to 1.0 × 10^5^ ± 1.0 × 10^4^ cfu ml^−1^ after 24 h. The bacteria survived intracellularly at 10^5^ cfu ml^−1^ for more than 2 weeks.

Beside the viable count assay, transmission electron microscopy was used to disclose intracellular localization of *V. mimicus* in *A. castellanii*. As samples of amoebae in absence and presence of *V. mimicus* were cut into ultra-thin sections to differentiate between intracellular and extracellular *V. mimicus*. According to the ultra-thin sections technique intracellular bacteria would be found inside the amoeba cells while extracellular bacteria will be found attached to the outside of amoeba cells. This should be compared with amoebae in the absence of bacteria, which accordingly have neither intracellular nor extracellular bacteria.

**Fig. 3:**
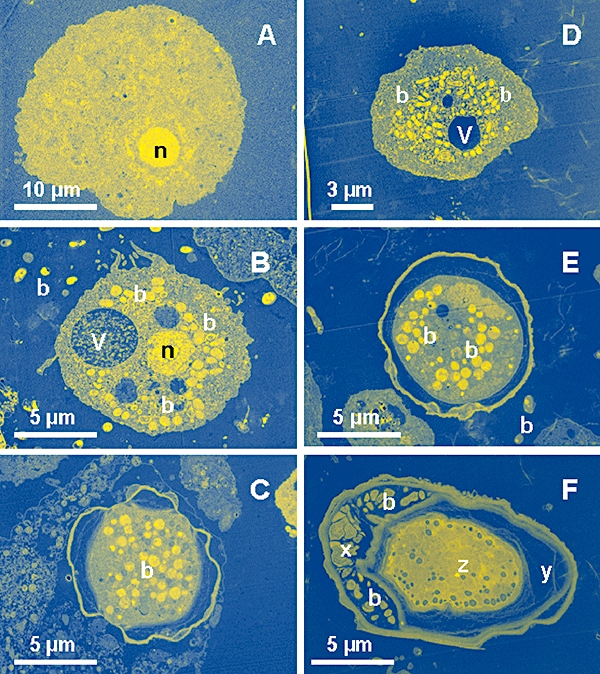
Electron microscopy of the intracellular localization of *V. mimicus* CS-5 in *A. castellanii*. b, bacteria; n, nucleus; v, vacuole; x, ectocyst; y, mesocyct; z, endocyst. A. *Acanthamoeba castellanii* trophozoite in absence of bacteria. B. *Vibrio mimicus* CS5 localized in cytoplasm of *A. castellanii* trophozoite, 1 day after co-cultivation. C. *Acanthamoeba castellanii* cyst contains intracellular *V. mimicus* CS5, 1 day after co-cultivation. D. *Acanthamoeba castellanii* trophozoite contains intracellular *V. mimicus* CS5, 3 days after co-cultivation. E. *Acanthamoeba castellanii* cyst contains intracellular *V. mimicus* CS5, 3 days after co-cultivation. F. *Acanthamoeba castellanii* precyst contains intracellular *V. mimicus* CS5 in space between ecto- and mesocyst, 3 days after co-cultivation.

Electron microscopy disclosed localization of co-cultivated *V. mimicus* in the cytoplasm of *A. castellanii* cells compared with amoebae in the absence of *V. mimicus* (Fig. 3A). Amoebae in the presence of wild *V. mimicus* showed that bacterial cells were located in the cytoplasm of amoeba trophozoite after 1 day of co-cultivation (Fig. 3B) and after 3 days of co-cultivation (Fig. 3D). The bacteria were also present in cysts after 1 day of co-cultivation (Fig. 3C) and after 3 days of co-cultivation (Fig. 3E). Moreover, the bacterial cells were found in space between ecto- and mesocyst of the precyst stage after 3 days of co-cultivation (Fig. 3F). The analysis also showed that the percentage of infected amoebae (trophozoite and cyst) was 57 ± 13 and that the number of bacteria inside amoeba cell was 37 ± 10.

This is the first report disclosing intracellular growth and survival of *V. mimicus* in *A. castellanii*. The bacteria grew intracellularly to 10^5^ cfu ml^−1^ on day 1 and survived intracellularly for more than 2 weeks. The current results showed that *V. mimicus* cells were localized in the cytoplasm of amoeba trophozoites. The bacteria were present in cysts and the intracellular bacteria were viable for more than 2 weeks. Thus, *V. mimicus* showed an intracellular behaviour in the acanthamoeba–host model.

Intracellular bacteria utilize different mechanisms to survive and multiply inside the host cells such as amoebae and macrophages. *Francisella tularensis* survives within the membrane-bounded vacuoles in macrophages (Greco *et al.*, 1987) as well as in *A. castellanii* (Abd *et al.*, 2003). *Shigella dysenteriae*, *Shigella sonnei*, *V. cholerae* O1 and O139 survive in the cytoplasm of *A. castellanii* trophozoites and bacteria can be found in cysts (Abd *et al.*, 2005; Abd *et al.*, 2007; Saeed *et al.*, 2009). Holden and colleagues (1984) found that 30–70% of *A. castellanii* contained intracellular *Legionella pneumophila*. In comparison, the present study shows that 40–70% of *A. castellanii* internalized *V. mimicus* and 25–55 bacteria could be counted per amoeba cell (Fig. 3D and F).

In contrast, extracellular bacteria are not able to grow in eukaryotic cells. It has been shown that *Acanthamoebae* benefit from the extracellular bacteria like *Escherichia coli* and *Klebsiella aerogenes* by using them as food (Weekers *et al.*, 1993), while the extracellular bacterium *P. aeruginosa* killed *A. castellanii* by the effect of type III-secreted proteins (Abd *et al.*, 2008). The reason for the difference in behaviour of different extracellular bacterial species is not known.

### Encystation of *A. castellanii* as a protective mechanism

Encystation of *A. castellanii* harbouring intracellular *V. mimicus* was performed to show that viability of the amoebae is not affected by the intracellular *V. mimicus* because the encystation is a vital process of the amoebic life cycle. Furthermore, to disclose another role of the cysts, this is to protect the intracellular bacteria from killing by antibiotics.

To examine the role of *Acanthamoeba* cysts to protect the intracellular bacteria from high level of gentamicin, *A. castellanii* cells were cultivated with *V. mimicus* CS-5 in ATCC 712 medium for 1 day, treated with 1000 µg ml^−1^ gentamicin to extracellular bacteria, recultivated in PBS and after the encystation process again treated with gentamicin. Viable counts were performed for the amoebae and intracellular *V. mimicus*.

The result of cell counts showed that the amoeba count was 1.6 × 10^5^ ± 5.7 × 10^3^ cfu ml^−1^ and that the all amoeba cells were cysts characterized by round cells with double walls having no nuclei at day 4. Viable counts of intracellular *V. mimicus* after treatment with 1000 µg ml^−1^ gentamicin was 2.5 × 10^4^ ± 7.0 × 10^3^ cfu ml^−1^ on day 4. These results clearly show that intracellularly harboured bacteria are protected from extracellular added gentamicin.

The ability of cysts to protect intracellular *V. mimicus* from gentamicin was investigated. In spite of susceptibility of *V. mimicus* to gentamicin (≤ 2 µg ml^−1^) determined by *E*-test growth of intracellular *V. mimicus* after 1000 µg ml^−1^ gentamicin treatment could be detected by viable count. Hence, cysts protected intracellular *V. mimicus* because gentamicin could not pass the double wall of the cysts.

*Vibrio mimicus* grows inside *A. castellanii* and the amoebae protected the bacterium from antibiotic killing. The outcomes of this interaction strongly point out the intracellular behaviour of *V. mimicus* as well as the ability of *A. castellanii* to host the bacterium in aquatic environments.

It has become increasingly apparent that bacterivorous predators such as free-living protozoa and nematodes could be exploited as model systems to gain significant insight into the pathogenesis of environmental bacteria (Hilbi *et al.*, 2007). *Vibrio mimicus* is primarily an extracellular enteropathogen, and thus it will be interesting to evaluate the status of the transcriptome of this bacterium during intracellular growth in *A. castellanii*. In this regard, our co-cultivation model will serve as a valuable tool. This warrants further investigation.
